# Neoadjuvant Versus Adjuvant Chemotherapy for Resectable Metastatic Colon Cancer in Non-academic and Academic Programs

**DOI:** 10.1093/oncolo/oyac209

**Published:** 2022-10-06

**Authors:** Zhonglin Hao, Saurabh Parasramka, Quan Chen, Aasems Jacob, Bin Huang, Timothy Mullett, Al B Benson

**Affiliations:** Department of Internal Medicine, Markey Cancer Center, University of Kentucky, Lexington, KY, USA; Department of Internal Medicine, Markey Cancer Center, University of Kentucky, Lexington, KY, USA; Biostatistics and Bioinformatics Shared Resource Facility, Markey Cancer Center, University of Kentucky, Lexington, KY, USA; Department of Internal Medicine, Markey Cancer Center, University of Kentucky, Lexington, KY, USA; Biostatistics and Bioinformatics Shared Resource Facility, Markey Cancer Center, University of Kentucky, Lexington, KY, USA; Division of Cancer Biostatistics, Department of Internal Medicine, University of Kentucky, Lexington, KY, USA; Department of Surgery, Markey Cancer Center, University of Kentucky, Lexington, KY, USA; Department of Medicine, Robert H. Lurie Comprehensive Cancer Center, Northwestern University, Chicago, IL, USA

**Keywords:** colon cancer, liver/lung metastasis, survival, neoadjuvant chemotherapy, adjuvant chemotherapy, surgery

## Abstract

**Background:**

Overall survival advantage of chemotherapy before versus after metastasectomy of liver or lung lesion is not clear for colon cancer with synchronous liver or lung metastasis.

**Materials and Methods:**

Adults 20 years or older with primary colon cancer and single organ metastatic disease either in the liver or lung at diagnosis were identified between 2010 and 2015 through the National Cancer Database (NCDB). Patients were categorized into 2 cohorts: pre-operative/peri-operative chemotherapy (neoadjuvant –[NAC]) or post-operative chemotherapy (adjuvant [AC]). Survivals and factors associated with were compared between the 2 groups.

**Results:**

A total of 3038 patients with colon cancer with liver or lung metastases were identified. The percentage of patients receiving NAC had steadily increased from 12.29% to 28.31%, mostly in academic programs. On multivariate analysis, patients who received NAC had an overall survival advantage in the non-academic setting whereas no advantage is seen in the patients treated in the academic settings. The median overall survival for patients receiving NAC and AC was 47.24 months and 38.08 months, respectively. Factors associated with overall survival advantage in NAC patients treated in non-academic programs included age 20-49 years, CEA value of >30, right-sided colon primary, liver metastasis, and clear resection margins.

**Conclusions:**

Metastatic colon cancer with single organ liver or lung lesions benefits from neoadjuvant chemotherapy, especially in ­non-academic settings. The overall survival advantage in this setting has not been shown before.

## Teaser

For resectable colon cancer with synchronous liver or lung metastasis, resection is recommended, along with pre/perioperative chemotherapy before or adjuvant chemotherapy after surgical resection of all lesions. This study compared survival outcome from both options retrospectively.

**Implications for Practice**

The OS benefit of NAC for patients with colon cancer with either synchronous liver or lung metastasis before surgical resection is not clear despite many theoretical advantages up to now. In the present study, a very clear overall survival benefit is shown in those who have received NAC. However, this advantage is limited to patients treated in settings other than academic suggesting the NAC approach may be able to compensate some of the inherent disadvantages leading to inferior survival when they are treated outside an academic setting. Therefore, NAC is highly recommended for this patient population in non-academic programs.

## Introduction

Approximately 50%-60% of all patients with colon cancer develop metastatic disease.^1-3^ Liver was the most common site of metastatic disease in approximately 50% patients, followed by lung in 20%.^4-6^ Most liver metastases are metachronous. However, an estimated 20%-34% patients have synchronous liver metastasis.^7,8^ It is estimated that more than half of the patients with colon cancer who die have liver metastasis and patients who were treated with liver resection had better survival than those who have not.^9^ In one series that analyzed 1001 patients, surgical resection of metastatic liver lesion resulted in a 5- and 10-year survival of 37% and 22%, respectively, while it ranged from 14%-74% and 9%-69%, respectively, in a retrospective review depending on the risk factors present.^4,10^ Retrospective analyses and meta-analyses revealed that patients who had solitary liver metastases and underwent resection had a 5-year overall survival rate of 71%.^11-13^ Hence, colon cancer with liver and/or lung metastasis is commonly resected aggressively with the goal of cure for many of these patients.^1,14^ Evidence also indicate that colon cancer with synchronous liver metastasis is associated with more disseminated states and poor outcome compared with metachronous liver metastasis.^15^

Neo-adjuvant chemotherapy (NAC) or adjuvant chemotherapy (AC) is thought to eradicate microscopic disease before and after resection. Also, chemotherapy can shrink tumors to enable R0 resections. Hence, it is incorporated into the treatment strategy for managing colon cancer with single organ metastasis to reduce the risk of recurrence and to increase cure rates. In a large database analysis of 82 609 patients treated between 2010 and 2015, multimodality treatment including systemic chemotherapy plus primary and metastatic site surgery (liver or lung) was performed in 8.2% of the patients and was associated with the greatest chance of survival.^16^ A prospective study of 364 patients with colon cancer and resectable 1-4 liver lesions showed that perioperative chemotherapy led to 7.3% absolute improvement in progression-free survival (PFS) at 3 years when compared with surgery alone, but only trended toward statistical significance (HR 0.79; 0.62-1.02; *P* = .058).^17^ A longer follow-up showed no difference of PFS. Importantly, median overall survival (mOS) was 61.3 months in the perioperative chemotherapy group vs. 54.3 months in the surgery alone group (HR 0.88, *P* = 0.34).^18^ Evidence for AC following resection of metastatic lesion(s) also comes from smaller randomized studies.^19-21^ Disease-free survival benefit of AC over surgery alone after resection ranged between 6.3% to 6.8%. Lately, AC after hepatectomy was shown to increase only 5-year DFS in the JCOG0603. DFS at 5 years were 38.7% and 49.8% in patients who were treated with hepatectomy only or hepatectomy plus chemotherapy respectively. There was no OS benefit shown in this trial.^22^

NAC converts disease to resectable status. In selected patients with unresectable liver lesions, induction chemotherapy may be used to convert it to resectable case. In a series of 1439 patients, 12.5% of the unresectable liver lesions became resectable with 5- and 10-year disease free survival (DFS) rate of 22% and 17%, respectively.^23^ In these situation, systemic treatment with chemotherapy, or immunotherapy for microsatellite instability (MSI) high tumors are rational choice.^24^

For colon cancer with synchronous liver or lung metastasis, National Comprehensive Cancer Network (NCCN) and European Society of Medical Oncology (ESMO) recommend upfront resection if complete resection (R0) is feasible; NAC before resection or AC after surgery are also acceptable choices. Perioperative/NAC has not been compared with AC in metastatic setting. We hypothesize that NAC confers an OS advantage over AC. Our study aimed at analyzing the National Cancer Database (NCDB) to examine the effect of treatment sequencing on overall survival of colon cancer patients with synchronous liver or lung metastasis treated by multidisciplinary approach within one year.

## Methods

### Data Source

NCDB is a nationally recognized database jointly sponsored by the American College of Surgeons and the American Cancer Society. It stores hospital-based de-identified data sourced from hospital cancer registries. Data are collected from more than 1500 Commission on Cancer (CoC)-accredited facilities and represents more than 72% of newly diagnosed cancer cases nationwide with more than 40 million historical records (https://www.facs.org/quality-programs/cancer/ncdb). As per our institution policy, a waiver for Institutional Review Board (IRB) approval was obtained since it was a database analysis of de-identified Health Insurance Portability and Accountability Act (HIPAA) compliant data file.

### Patient Selection and Method

We identified patients 20 years and older with colon adenocarcinoma from year 2004-2016 using International Classification of Diseases (ICD)-O-3 morphological codes 8000-8152, 8154-8231, 8243-8245, 8247, 8248, 8250-8934, 8940-9136, 9141-9582, 9700-9701 and topographical codes from C18.0 to C18.9.

Patients diagnosed between 2004 and 2009 were excluded as American Joint Committee on Cancer (AJCC) 6th edition staging did not differentiate between single and multiple organ metastases. Patients diagnosed between 2010 and 2015 with AJCC 7th edition staging classification were eligible as it differentiates between number and types of organs involved by metastatic disease. Patients were also limited to those who had metastatic disease to only liver or lung. Resection of metastatic sites was thought to have happened if patients underwent “resection of distant site” along with “resection of primary site” in any order and were eligible for inclusion. Surgical approach (ie, staged surgery versus simultaneous primary and metastasectomy versus liver first or lung first approach) was not identified. A recent paper published on this topic identified only 2% of patients had liver first approach and 98% had simultaneous or staged surgery.^25^ NCDB data dictionary specifies receipt of chemotherapy includes at least 2 courses either pre- or post- operatively. Based upon treatment, patients were divided into 2 cohorts one which received preoperative chemotherapy followed by surgery, with or without post-operative chemotherapy- NAC group and another cohort which received post-operative chemotherapy only—AC group. All treatments were delivered within 1 year from the time of diagnosis.

We extracted patient information including age, sex, race, Charlson/Deyo comorbidity index (CCI), income, insurance, and facility information. Disease-related characteristics included histology, differentiation, sidedness, CEA at the time of diagnosis, clinical and pathological TNM staging, circumferential resection margin (CRM) status, KRAS status, and microsatellite instability status (MSI). Cancer Centers were divided into academic and non-academic centers based on NCDB definition. Community Cancer Program (CCP), Comprehensive Community Cancer Program (CCCP), and Integrated Cancer Program (INCP) were classified as non-academic centers, and Academic Research Program (ARP) was classified as academic. The “Other” category in NCDB included both academic and non-academic institutions and were excluded from center-based analysis.

### Statistical Analysis

Overall survival (OS) was defined as time in months from diagnosis to either death or last follow-up date. Descriptive analysis was performed to examine the characteristics of clinical and demographics variables by the 2 treatment procedures. Pearson’s Chi-square tests were used to identify statistical significances. Similar analysis was done for comparing academic setting verse nonacademic setting. Kaplan-Meier (KM) plots were used to examine survival curves by treatment types and academic status. Cox proportional hazard model was used to examine the impact of metastasis site and treatment type on OS, while adjusting for patient demographic, clinical factors. All statistical tests were 2 sided. A significant level of statistical significance was defined as a *P*-value of <.05 and analysis was conducted utilizing SAS 9.4 (Cary, NC).

## Results

### Baseline Characteristics and Treatment Trends

**Figure 1.**
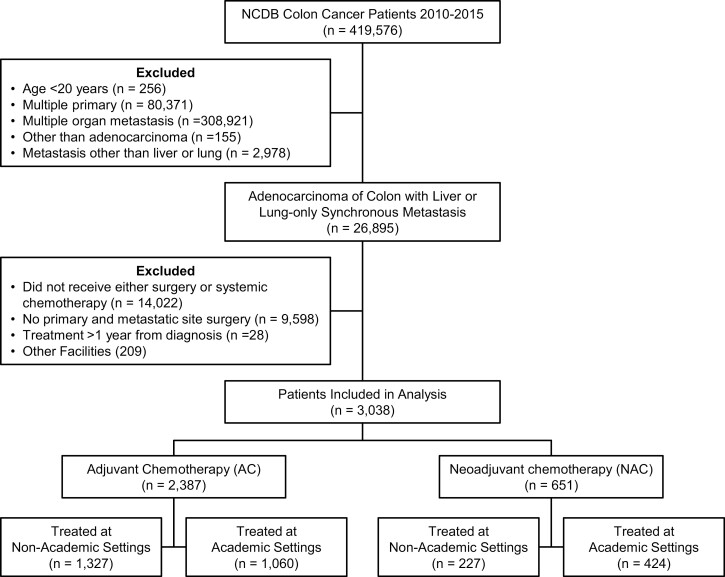
CONSORT diagram.

**Figure 2.**
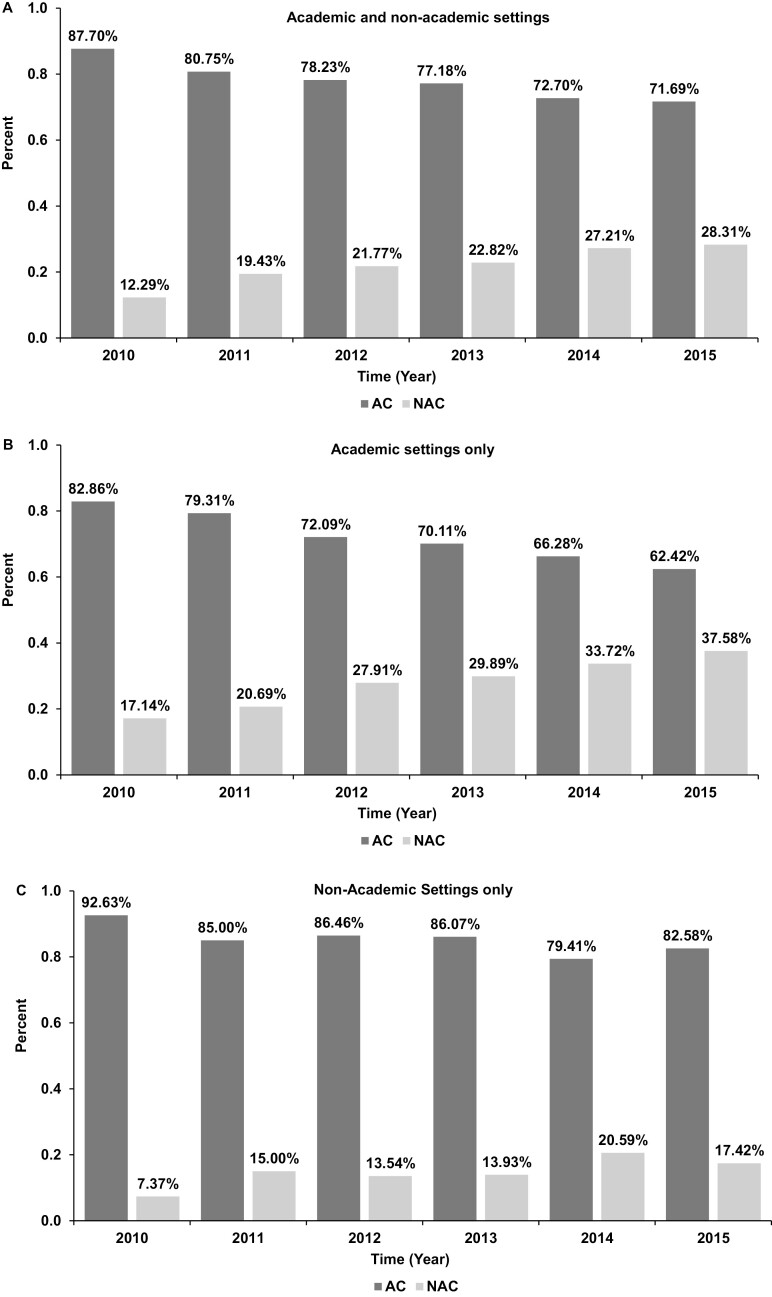
ABC. Neoadjuvant and adjuvant chemo in academic vs non-academic programs (trend).

Between 2010 and 2015, there were 26 895 patients who had diagnosis of colon cancer with liver or lung-only metastasis. Among them, 3038 patients underwent surgery to metastatic (liver or lung) sites and received NAC or AC (Fig. 1). Resection of liver-only metastasis was performed in 2958 patients while lung-only metastasis resection was performed only in 80 patients. Surgery first followed by postoperative chemotherapy (AC) approach was used in 2387 (78.3%) patients and chemotherapy first (NAC) followed by surgery in the remaining 651 (21.7%) patients. Table 1 summarizes the baseline characteristics of patients in both cohorts. Patients in the NAC group were younger than the AC group. Median age of the study population was 56 years for the NAC group and 59 years for the AC group. Eighty percent of the patients were Caucasians and 54% male in both groups. Less than 5% patients had CCI score of 2 or more in either group. In the AC group, median time from diagnosis to surgery was 7 days and diagnosis to start of chemotherapy was 56 days. In the NAC group, the median time from diagnosis to chemotherapy was 26 days and diagnosis to surgery was 157.5 days. Roughly equal number of patients were treated in academic and non-academic programs during this period (1484 vs 1554). Twice as many patients in academic programs received NAC in the same period (28.57% vs 14.61%). Patients in the AC group had more left sided tumors (50.7% vs. 42.4%), but more poorly differentiated cancer (16.4% vs. 8.4%). NAC group, however, had higher likelihood of having preoperative CEA >30 ng/mL (32.9% vs 22.4%). As expected, the NAC group had fewer positive circumferential resection margin compared to the AC (4.5% vs 10.8%). Although the number of regional nodes examined are not different between patients who have received AC or NAC in both academic and non-academic programs (*P* = .5095 and .3097, respectively), significantly more regional lymph nodes were found positive in the AC compared wit the NAC patients in both academic and non-academic programs (Table 1). As presented in Fig. 2, there was a clear trend of increased use of NAC over the study period, with proportion of patients receiving NAC increased from 12.3% of all patients in 2010 to 28.3% in 2015 in all programs. While NAC use saw a steady increase over the years reaching 37.6% of all patients seen in academic programs in 2015, NAC use in non-academic programs stagnated around 15 to 20% after initial doubling from 7.37% in 2010 to 15% in 2011. The proportion of patients receiving NAC in non-academic programs is barely half that of academic programs in 2015.

**Table 1.** Patient characteristics by treatment type.

Category	Variable	AC (%)	NAC (%)	*P*-value
Total		2387	651	
Age group	20-49	379 (15.9)	131 (20.1)	.0131
	50-64	1115 (46.7)	315 (48.4)	
	65-74	621 (26)	143 (22)	
	75+	272 (11.4)	62 (9.5)	
Race	White	1909 (80)	524 (80.5)	.0002
	Black	382 (16)	81 (12.4)	
	Other	78 (3.3)	43 (6.6)	
	Unknown	18 (0.8)	3 (0.5)	
Gender	Male	1308 (54.8)	355 (54.5)	.904
	Female	1079 (45.2)	296 (45.5)	
Year of diagnosis	2010-2012	1196 (50.1)	235 (36.1)	<.0001
	2013-2015	1191 (49.9)	416 (63.9)	
Metastatic site	Liver	2314 (96.9)	644 (98.9)	.0051
	Lung	73 (3.1)	7 (1.1)	
Grade	Well Differentiated	109 (4.6)	25 (3.8)	<.0001
	Moderately Differentiated	1717 (71.9)	467 (71.7)	
	Poorly Differentiated	392 (16.4)	55 (8.4)	
	Undifferentiated	77 (3.2)	10 (1.5)	
	Unknown	92 (3.9)	94 (14.4)	
CCI	0	1832 (76.7)	508 (78)	.6871
	1	431 (18.1)	114 (17.5)	
	≥2	124 (5.2)	29 (4.5)	
Sidedness	C180-184 (left)	1211 (50.7)	276 (42.4)	.0002
	C185-187 (right)	1090 (45.7)	357 (54.8)	
	C188,189,260 (unknown)	86 (3.6)	18 (2.8)	
CEA	<30.0	1045 (43.8)	288 (44.2)	<.0001
	≥30.0	535 (22.4)	214 (32.9)	
	Unknown	807 (33.8)	149 (22.9)	
MSI	Negative	626 (26.2)	191 (29.3)	.2253
	Positive	68 (2.8)	21 (3.2)	
	Unknown	1693 (70.9)	439 (67.4)	
CRM	≤10	310(13.0)	31(4.8)	
	10+	549(23.0)	136(20.9)	<.0001
	Unknown	1528(64.0)	484(74.3)	
KRAS	Negative	606 (25.4)	187 (28.7)	.0095
	Positive	513 (21.5)	162 (24.9)	
	Unknown	1268 (53.1)	302 (46.4)	
Surgery margin	Negative	2045 (85.7)	608 (93.4)	<.0001
	Positive	257 (10.8)	29 (4.5)	
	Unknown	85 (3.6)	14 (2.2)	
Facility type	Non-academic	1327 (55.6)	227 (34.9)	<.0001
	Lymph nodes examined (mean ± SD)	20.3 ± 10.6	19.5 ± 10.5	.3097
	Lymph nodes positive (mean ± SD)	4.3 ± 4.8	1.7 ± 2.7	<.0001
	Academic	1060 (44.4)	424 (65.1)	
	Lymph nodes examined (mean ± SD)	21.5 ± 11.3	21.1 ± 11.7	.5095
	Lymph nodes positive (mean ± SD)	3.8 ± 4.2	2.1 ± 3.0	<.0001

Abbreviations: NAC, neoadjuvant chemotherapy; AC, adjuvant chemotherapy; CCI, Charlson Comorbidity Index; CEA, carcinoembryonic antigen; MSI, microsatellite instability; KRAS, Kirsten rat sarcoma viral oncogene homolog.

### Survival Analysis

**Figure 3.**
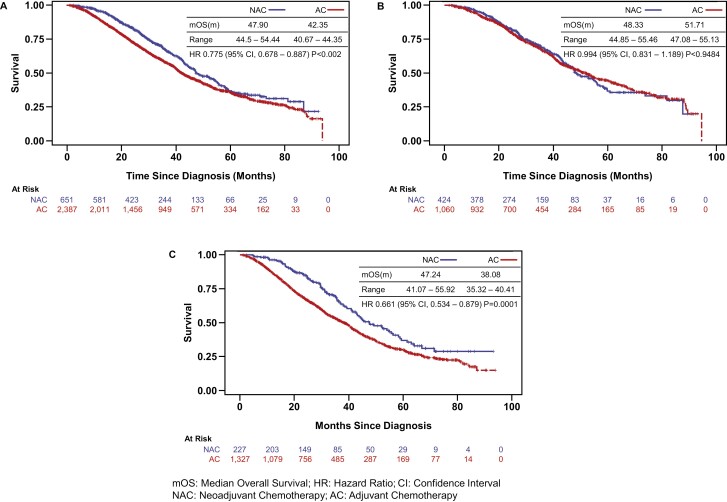
ABC. Survival curve of patients with different sequence of treatment (NAC vs AC).

**Figure 4.**
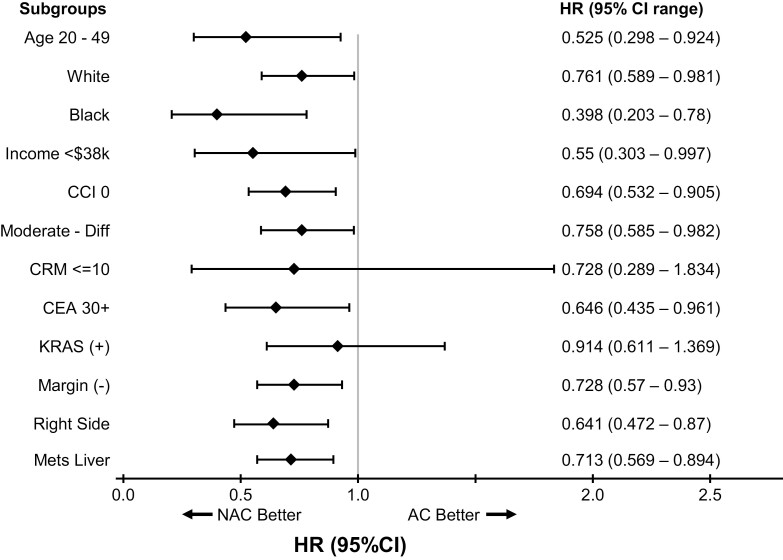
Mortality hazard ratios according to baseline co-variants in non-academic programs.

Given this potential advantage of NAC over AC in outcome, we set to analyze if survival for patients treated with NAC was different from those treated with AC. Kaplan-Meier survival curves were plotted comparing the NAC and AC groups inclusive of patients from all programs. As shown in Fig. 3A, patients with colon cancer with single organ metastases to liver or lung when treated with pre-/peri-operative chemotherapy followed by surgery had significantly better overall survival when compared to the group treated with surgery followed by chemotherapy. mOS for NAC and AC were 47.90 months and 42.35 months, respectively (HR 0.775; 95% CI 0.678-0.887; *P* < .002). Considering the advantage of patients treated in high volume academic center and commonly by a multi-disciplinary team, we decided to test the interaction term between treatment types and programs where patients were treated. And the result was significant. Therefore, we performed the survival analysis in 2 different cohorts: academic programs vs non-academic programs. Patient characteristics by treatment facilities is shown in Table 2. NAC did not convey any survival advantage over AC in patients treated in academic programs (mOS 48.33m vs 51.71m, HR 0.994; 95% CI 0.831-1.189; *P* < .9484; Fig. 3B). In comparison, median survival is much longer for patients with NAC than for patients with AC in non-academic programs (mOS 47.24m vs. 38.08m, HR 0.661; 95%CI 0.534-0.879; *P* < .0001) (Fig. 3C). Table 3 shows 3 different Cox regression models constructed, showing factors predicting survival outcomes (M1-3). For patients treated in academic programs (M2), NAC is no different from AC. For patients treated in non-academic programs, NAC is better than AC (HR 0.71) (M3). Factors associated with poor prognosis include older age (>75 years, HR 1.72); CCI of 2 or above (HR 1.63); CEA 30 or higher (HR 1.59) and positive surgical margin (HR 1.51). Left-sided colon cancer is a better prognostic factor (HR 0.714). Factors associated with poor prognosis include annual income less than $47 999 (HR 1.19-1.81); poorly differentiated or undifferentiated tumor (HR 1.97). Further analysis showed that factors associated with better outcome, when patients receive NAC, include age 20-49 years; income less than $38,000; CCI 0; moderate differentiation; CEA level 30 or above; negative surgical margin status; right sided colon and metastasis to the liver. (Fig. 4). For patients treated in the academic program, no differences were seen (data not shown).

**Table 2.** Patient characteristics by treatment facility.

Category	Variable	Non-academic (%)	Academic (%)	*P*-value
Total		1554	1484	
Age group	20-49	242 (15.6)	268 (18.1)	.0028
	50-64	709 (45.6)	721 (48.6)	
	65-74	405 (26.1)	359 (24.2)	
	75+	198 (12.7)	136 (9.2)	
Race	White	1264 (81.3)	1169 (78.8)	.0152
	Black	235 (15.1)	228 (15.4)	
	Other	49 (3.2)	72 (4.9)	
	Unknown	6 (0.4)	15 (1)	
Gender	Male	839 (54)	824 (55.5)	.3952
	Female	715 (46)	660 (44.5)	
Year of diagnosis	2010-2012	774 (49.8)	657 (44.3)	.0023
	2013-2015	780 (50.2)	827 (55.7)	
Metastatic site	Liver	1498 (96.4)	1460 (98.4)	.0006
	Lung	56 (3.6)	24 (1.6)	
Grade	Well differentiated	82 (5.3)	52 (3.5)	<.0001
	Moderately differentiated	1099 (70.7)	1085 (73.1)	
	Poorly differentiated	229 (14.7)	218 (14.7)	
	Undifferentiated	67 (4.3)	20 (1.3)	
	Unknown	77 (5)	109 (7.3)	
CCI	0	1169 (75.2)	1171 (78.9)	.0158
	1	292 (18.8)	253 (17)	
	≥2	93 (6)	60 (4)	
Sidedness	Left	814 (52.4)	673 (45.4)	.0004
	Right	686 (44.1)	761 (51.3)	
	Overlapping and Unknown	54 (3.5)	50 (3.4)	
CEA	<30.0	690 (44.4)	643 (43.3)	.4002
	≥30.0	392 (25.2)	357 (24.1)	
	Unknown	472 (30.4)	484 (32.6)	
MSI	Negative	387 (24.9)	430 (29)	.0402
	Positive	46 (3)	43 (2.9)	
	Unknown	1121 (72.1)	1011 (68.1)	
	≤10	209 (13.5)	132 (8.9)	<.0001
CRM	10+	429 (27.6)	256 (17.3)	
	Unknown	916 (58.9)	1096 (73.9)	
KRAS	Negative	438 (28.2)	355 (23.9)	.0247
	Positive	341 (21.9)	334 (22.5)	
	Unknown	775 (49.9)	795 (53.6)	
Surgery margin	Negative	1328 (85.5)	1325 (89.3)	.0032
	Positive	173 (11.1)	113 (7.6)	
	Unknown	53 (3.4)	46 (3.1)	

Abbreviations: NAC, neoadjuvant chemotherapy; AC, adjuvant chemotherapy; CCI, Charlson Comorbidity Index; CEA, carcinoembryonic antigen; MSI, microsatellite instability; KRAS, Kirsten rat sarcoma viral oncogene homolog.

**Table 3.** Cox regression model.

		M1-academic and non-academic		M2-academic		M3-non-academic	
		Hazard ratio	*P*-value	Hazard ratio	*P*-value	Hazard ratio	*P*-value
Treatment	AC	Ref		—		——	.003
	NAC	0.86 (0.75-0.99)	.039	1.03 (0.85-1.24)	.782	0.71 (0.57-0.89)	
Academic	Yes	Ref	<.001				
	No	1.31 (1.18-1.46)					
Age group	50-64	Ref	<.001	—	.033	—	<.001
	20-49	1.10 (0.95-1.28)		1.15 (0.93-1.44)		1.09 (0.89-1.34)	
	65-74	1.26 (1.11-1.43)		1.13 (0.93-1.38)		1.37 (1.16-1.62)	
	75+	1.61 (1.38-1.89)		1.48 (1.13-1.93)		1.72 (1.40-2.11)	
Race	White	Ref	.191	—	.67	—	.133
	Black	1.04 (0.89-1.20)		0.97 (0.77-1.23)		1.10 (0.90-1.35)	
	Other	1.03 (0.78-1.38)		0.86 (0.58-1.28)		1.28 (0.84-1.95)	
	Unknown	0.39 (0.16-0.94)		0.60 (0.22-1.61)		0.15 (0.02-1.11)	
Median income quartiles	≥$63 000	Ref	.004	—	.743	—	.003
	$38 000-$47 999	1.19 (1.04-1.38)		1.11 (0.89-1.38)		1.30 (1.07-1.57)	
	$48 000-$62 999	1.14 (0.99-1.31)		1.02 (0.83-1.25)		1.25 (1.04-1.51)	
	<$38 000	1.34 (1.15-1.57)		1.15 (0.91-1.46)		1.51 (1.22-1.86)	
	Not available	1.04 (0.33-3.24)		1.36 (0.19-10.00)		0.85 (0.21-3.47)	
CCI	0	Ref	<.001	—	.022	—	.001
	1	1.03 (0.91-1.18)		0.97 (0.79-1.20)		1.09 (0.92-1.29)	
	≥2	1.63 (1.31-2.01)		1.69 (1.16-2.47)		1.63 (1.26-2.12)	
Grade	Well differentiated	Ref	<.001	—	.027	—	<.001
	Unknown	0.75 (0.53-1.04)		0.87 (0.52-1.47)		0.66 (0.41-1.06)	
	Moderately differentiated	0.85 (0.66-1.08)		0.84 (0.55-1.27)		0.86 (0.64-1.17)	
	Poorly differentiated	1.34 (1.03-1.74)		1.15 (0.74-1.79)		1.53 (1.10-2.14)	
	Undifferentiated	1.97 (1.39-2.78)		1.43 (0.68-3.03)		2.19 (1.46-3.28)	
CEA	<30.0	Ref					<.001
	≥30.0	1.59 (1.40-1.80)	<.001	1.51 (1.24-1.84)	<.001	1.59 (1.35-1.88)	
	Unknown	1.08 (0.96-1.22)		1.10 (0.91-1.34)		1.05 (0.89-1.24)	
Surgery margins	Margin negative	Ref					.002
	Positive	1.39 (1.15-1.68)	<.001	1.22 (0.89-1.67)	.218	1.51 (1.18-1.92)	
	Unknown	1.32 (0.99-1.76)		1.32 (0.85-2.06)		1.33 (0.90-1.95)	
CRM	10+	Ref					.093
	≤10	1.10 (0.90-1.34)	<.001	1.30 (0.93-1.80)	<.001	1.00 (0.77-1.29)	
	Unknown	0.80 (0.70-0.91)		0.72 (0.58-0.90)		0.85 (0.72-1.00)	
KRAS	Negative	Ref	.011		.011		.260
	Positive	1.22 (1.05-1.42)		1.35 (1.07-1.70)		1.16 (0.95-1.41)	
	Unknown	1.02 (0.90-1.16)		1.02 (0.83-1.25)		1.01 (0.86-1.19)	
Mets to another Site	Liver	Ref	.018		.148		.066
	Lung	0.64 (0.44-0.93)		0.52 (0.21-1.26)		0.68 (0.45-1.03)	
Sidedness	Right	Ref					<.001
	Left	0.75 (0.67-0.84)	<.001	0.78 (0.66-0.93)	.013	0.71 (0.61-0.82)	
	Other	1.04 (0.79-1.36)		1.05 (0.69-1.60)		1.01 (0.70-1.44)	

## Discussion

Current NCCN guidelines encourage a course of systemic treatment for most patients with metastatic colon cancer planned to have liver or lung resection to increase the chance to eradicate microscopic disease. For resectable disease, both AC after resection and NAC (including perioperative chemotherapy given before and after surgery) are acceptable. Evidence supporting AC after resection of cancer come from studies showing PFS benefit in 642 patients in one meta-analysis of 3 clinical trials in 2012.^26^ JCOG0603 randomized patients with colorectal cancer with liver metastasis only to liver resection alone or liver resection followed by adjuvant chemotherapy. DFS at 5 years was improved in the chemotherapy group (49.8% vs 38.7%). However, there was no OS benefit (71.2% vs 83.1%).^22^ Another meta-analysis of 1896 patients from 10 trials also found improved DFS in patients receiving perioperative chemotherapy in 2015.^27^ Similarly, EORTC 40983, which compared perioperative chemotherapy and liver resection with resection alone in patients with colorectal cancer liver metastasis, showed only PFS benefit of adding perioperative chemotherapy, and there was no OS benefit.^18^ Both studies failed to show an OS benefit in these patients. However, due to the lack of evidence, the optimal sequencing of systemic therapy and surgical resection is not clear.

Adjuvant chemotherapy given before or both before and after surgery (NAC) is considered to have many advantages over AC. These include potential to convert to resectable disease or have more R0 resection when it is difficult to judge the resectability upfront; clear up occult metastasis including in the lymph nodes early; as well as assessing chemosensitivity and biology of the disease and a chance to adjust chemotherapy regimen. In addition, treatment systemically help avoid futile local treatment when disease progresses early in the course. Compared to AC, NAC is much easier to give and less prone to complication. Earlier studies employing the surgery first followed by chemotherapy and metastasectomy approach were frequently associated with failure to complete course because nearly half of them never made it to surgery likely due to chemotherapy delay and disease progression.^28,29^ Interestingly, a recent report showed that preoperative systemic chemotherapy alters the histopathological growth patterns (HPG) of colorectal cancer liver metastasis. Chemotherapy changes the tumor HPG to a desmoplastic type which reflects good prognosis.^30^ Such change was significantly more in the EORTC 40983 liver resection specimens receiving perioperative chemotherapy compared with liver resection alone (61% vs 33%). Although NAC has its disadvantages which include potential disease progression during systemic treatment thus missing the opportunity of resection and adverse effects on organ function such as liver steatohepatitis and sinusoidal liver injury in patients receiving oxaliplatin or irinotecan,^31-34^ we nonetheless hypothesized that NAC in patients with synchronous liver or lung metastasis contain systemic disease early and provide the best chance of survival when the primary and metastatic tumor are resected eventually.

Our study here compared sequencing of chemotherapy with surgery on patient overall survival when multimodality treatment with surgery at the primary and metastatic site with chemotherapy given in the pre-/perioperative or postoperative setting. In this retrospective analysis involving 3038 patients treated between 2010 and 2015, we found that chemotherapy given pre- or peri-operatively improved overall survival. Interestingly, this advantage is clear only in patients treated in programs other than academic ones. The magnitude of benefit is very significant with HR of 0.661, *P* = .0001 among patients treated in non-academic programs. Within the academic programs, however, the NAC approach is no different from the AC approach, supporting current recommendation from the guideline when patients are seen in academic programs. While the lack of benefit of NAC over AC in academic programs can potentially be explained by near maximum use of NAC in these programs, this is clearly not the case in non-academic programs (Fig. 2). The overall survival advantage of NAC over AC may reflect 2 things. First, NAC shrinks the tumor before surgery, so it is easier to get a clean resection margin. NAC converts more de facto unresectable case in the beginning to achieve a R0 resection.^35,36^ In programs where no regular multidisciplinary tumor board review, case selection to determine resectability is perceived to be more difficult. We know that T4b tumors benefit from NAC than any other situation.^37^ If this is true, then data would show higher percentage of positive resection margins in patients treated with AC compared to NAC. Positive margin rate is indeed much higher in AC patients compared to NAC patients (Δ+6.3%, Table 2). Second, NAC may sterilize more regional lymph nodes before resection. Our results indeed shows that more nodes were found involved in AC compared with NAC while similar number of lymph nodes were dissected during surgery between the 2 groups, Table 1. Despite all the disadvantages compared to an academic program, NAC was able to compensate all these in the non-academic settings. Indeed, those patients who were treated in a non-academic program with NAC had survival comparable to those in the academic programs with a mOS of 47 months (Fig. 3).

The use of NAC in non-academic programs however was suboptimal compared to the use in academic programs, which more than doubled in 6 years. NAC use in non-academic program is less than half of that in academic programs from 2011 to 2015 (17.14% vs 37.58%). It is worth noting here NAC does not improve overall survival in academic cancer programs, but it does so in non-academic programs in our analysis. Therefore, continued awareness education to oncologists practicing in the non-academic setting is expected to make a difference in survival in these patients. Caution is advised to interpret the statistically insignificant survival outcome between NAC and AC in academic programs. NAC did result in more nodes being sterilized (Table 1) in academic programs. Our hypothesis for the “lack of overall survival benefit of NAC over AC in academic programs” is that this is a selection bias. That is: we probably have assigned all patients with potential benefit with NAC to the NAC group in academic programs. As a result, all patients with AC were best suited for upfront resection therefore had the best survival possible without upfront chemotherapy.

## Strengths and Limitations

NCDB is a large hospital-based database. Even with such a narrow population of interest, we were able to identify over 3000 patients. It is not possible to study this question in a prospective fashion in this number. Information was collected from both non-academic and academic programs mimicking the real-world situation. We show here an overall survival advantage of systemic chemotherapy pre-/perioperatively over postoperatively when added on to resection of both the primary and the metastatic colon cancer. However, this is helpful only in non-academic programs, which is unprecedented. Being a retrospective database study there is a risk of selection bias that could be hard to mitigate. There are also certain pieces of information which are not included as part of the database in NCDB; eg, it does not have information about the number and size of lesions in the metastatic organ which has important prognostic implication. It does not give details about chemotherapy agents, number of cycles of chemotherapy given therefore difficult to determine the benefit of chemotherapy in general. There is no information on *BRAF* mutational status neither.

## Conclusion

Neoadjuvant chemotherapy or perioperative chemotherapy seems to be under-utilized in metastatic colon cancer with synchronous liver or lung metastasis when seen in a non-academic program. Sequencing it before definitive resection of primary and metastatic site improves overall survival and should be encouraged in non-academic programs. Prospective, randomized clinical trials conducted in academic and non-academic programs; comparing neoadjuvant to adjuvant, is expected to provide robust evidence regarding the best practice in these situations. The trial will surely need to incorporate emerging approach in monitoring of minimal residual disease by circulating tumor DNA.

## Data Availability

The data underlying this article will be shared on reasonable request to the corresponding author.

## References

[1] Van Cutsem E , Nordlinger B, Adam R, et al. Towards a pan-European consensus on the treatment of patients with colorectal liver metastases. Eur J Cancer. 2006;42(14):2212-2221. 10.1016/j.ejca.2006.04.012. 16904315

[2] Alberts SR , Horvath WL, Sternfeld WC, et al. Oxaliplatin, fluorouracil, and leucovorin for patients with unresectable liver-only metastases from colorectal cancer: a North Central Cancer Treatment Group phase II study. J Clin Oncol. 2005;23(36):9243-9249. 10.1200/JCO.2005.07.740. 16230673

[3] Lee WS , Yun SH, Chun HK, et al. Pulmonary resection for metastases from colorectal cancer: prognostic factors and survival. Int J Colorectal Dis. 2007;22(6):699-704. 10.1007/s00384-006-0218-2. 17109105

[4] Kanas GP , Taylor A, Primrose JN, et al. Survival after liver resection in metastatic colorectal cancer: review and meta-analysis of prognostic factors. Clin Epidemiol 2012;4(1):283-301. 10.2147/CLEP.S34285. 23152705 PMC3496330

[5] Abdalla EK , Adam R, Bilchik AJ, et al. Improving resectability of hepatic colorectal metastases: expert consensus statement. Ann Surg Oncol. 2006;13(10):1271-1280. 10.1245/s10434-006-9045-5. 16955381

[6] Galandiuk S , Wieand HS, Moertel CG, et al. Patterns of recurrence after curative resection of carcinoma of the colon and rectum. Surg Gynecol Obstet 1992;174(1):27-32. 1729745

[7] Muratore A , Zorzi D, Bouzari H, et al. Asymptomatic colorectal cancer with un-resectable liver metastases: immediate colorectal resection or up-front systemic chemotherapy?. Ann Surg Oncol. 2007;14(2):766-770. 10.1245/s10434-006-9146-1. 17103261

[8] Hayashi M , Inoue Y, Komeda K, et al. Clinicopathological analysis of recurrence patterns and prognostic factors for survival after hepatectomy for colorectal liver metastasis. BMC Surg. 2010;10(2):1-12. 10.1186/1471-2482-10-27. 20875094 PMC2949597

[9] Foster JH. Treatment of metastatic disease of the liver: a skeptic’s view. Semin Liver Dis. 1984;4(2):170-179. 10.1055/s-2008-1040656. 6205450

[10] Fong Y , Fortner J, Sun RL, Brennan MF, Blumgart LH. Clinical score for predicting recurrence after hepatic resection for metastatic colorectal cancer: analysis of 1001 consecutive cases. Ann Surg. 1999;230(3):309-18; discussion 318. 10.1097/00000658-199909000-00004. discussion 1821. 10493478 PMC1420876

[11] Aloia TA , Vauthey JN, Loyer EM, et al. Solitary colorectal liver metastasis: resection determines outcome. Arch Surg. 2006;141(5):460-6; discussion 466. 10.1001/archsurg.141.5.460. discussion 67. 16702517

[12] Hur H , Ko YT, Min BS, et al. Comparative study of resection and radiofrequency ablation in the treatment of solitary colorectal liver metastases. Am J Surg. 2009;197(6):728-736. 10.1016/j.amjsurg.2008.04.013. 18789428

[13] Lee WS , Yun SH, Chun HK, et al. Clinical outcomes of hepatic resection and radiofrequency ablation in patients with solitary colorectal liver metastasis. J Clin Gastroenterol. 2008;42(8):945-949. 10.1097/MCG.0b013e318064e752. 18438208

[14] Charnsangavej C , Clary B, Fong Y, et al. Selection of patients for resection of hepatic colorectal metastases: expert consensus statement. Ann Surg Oncol. 2006;13(10):1261-1268. 10.1245/s10434-006-9023-y. 16947009

[15] Tsai MS , Su YH, Ho MC, et al. Clinicopathological features and prognosis in resectable synchronous and metachronous colorectal liver metastasis. Ann Surg Oncol. 2007;14(2):786-794. 10.1245/s10434-006-9215-5. 17103254

[16] Zhang GQ , Taylor JP, Stem M, et al. Aggressive multimodal treatment and metastatic colorectal cancer survival. J Am Coll Surg. 2020;230(4):689-698. 10.1016/j.jamcollsurg.2019.12.024. 32014570

[17] Nordlinger B , Sorbye H, Glimelius B, et al. Perioperative chemotherapy with FOLFOX4 and surgery versus surgery alone for resectable liver metastases from colorectal cancer (EORTC Intergroup trial 40983): a randomised controlled trial. Lancet 2008;371(9617):1007-1016. 10.1016/S0140-6736(08)60455-9. 18358928 PMC2277487

[18] Nordlinger B , Sorbye H, Glimelius B, et al. Perioperative FOLFOX4 chemotherapy and surgery versus surgery alone for resectable liver metastases from colorectal cancer (EORTC 40983): long-term results of a randomised, controlled, phase 3 trial. Lancet Oncol. 2013;14(12):1208-1215. 10.1016/S1470-2045(13)70447-9. 24120480

[19] Portier G , Elias D, Bouche O, et al. Multicenter randomized trial of adjuvant fluorouracil and folinic acid compared with surgery alone after resection of colorectal liver metastases: FFCD ACHBTH AURC 9002 trial. J Clin Oncol. 2006;24(31):4976-4982. 10.1200/JCO.2006.06.8353. 17075115

[20] Ychou M , Hohenberger W, Thezenas S, et al. A randomized phase III study comparing adjuvant 5-fluorouracil/folinic acid with FOLFIRI in patients following complete resection of liver metastases from colorectal cancer. Ann Oncol. 2009;20(12):1964-1970. 10.1093/annonc/mdp236. 19567451

[21] Hasegawa K , Saiura A, Takayama T, et al. Adjuvant oral uracil-tegafur with leucovorin for colorectal cancer liver metastases: a randomized controlled trial. PLoS One. 2016;11(9):e0162400. 10.1371/journal.pone.0162400. 27588959 PMC5010179

[22] Kanemitsu Y , Shimizu Y, Mizusawa J, et al. Hepatectomy followed by mFOLFOX6 versus hepatectomy alone for liver-only metastatic colorectal cancer (JCOG0603): a phase II or III randomized controlled trial. J Clin Oncol. 2021;39(34):3789-3799. 10.1200/JCO.21.01032. 34520230

[23] Adam R , Delvart V, Pascal G, et al. Rescue surgery for unresectable colorectal liver metastases downstaged by chemotherapy: a model to predict long-term survival. Ann Surg. 2004;240(4):644-57; discussion 657. 10.1097/01.sla.0000141198.92114.f6. discussion 578. 15383792 PMC1356466

[24] Coukos G. Neoadjuvant immune-checkpoint blockade in resectable colon cancer. Nat Med. 2020;26(4):473-474. 10.1038/s41591-020-0826-3. 32251401

[25] Kurbatov V , Resio BJ, Cama CA, et al. Liver-first approach to stage IV colon cancer with synchronous isolated liver metastases. J Gastrointest Oncol 2020;11(1):76-83. 10.21037/jgo.2020.01.03. 32175108 PMC7052756

[26] Ciliberto D , Prati U, Roveda L, et al. Role of systemic chemotherapy in the management of resected or resectable colorectal liver metastases: a systematic review and meta-analysis of randomized controlled trials. Oncol Rep. 2012;27(6):1849-1856. 10.3892/or.2012.1740. 22446591

[27] Wang ZM , Chen YY, Chen FF, Wang SY, Xiong B. Peri-operative chemotherapy for patients with resectable colorectal hepatic metastasis: a meta-analysis. Eur J Surg Oncol. 2015;41(9):1197-1203. 10.1016/j.ejso.2015.05.020. 26094113

[28] Yoshidome H , Kimura F, Shimizu H, et al. Interval period tumor progression: does delayed hepatectomy detect occult metastases in synchronous colorectal liver metastases?. J Gastrointest Surg. 2008;12(8):1391-1398. 10.1007/s11605-008-0540-9. 18491195

[29] Collins D , Chua H. Contemporary surgical management of synchronous colorectal liver metastases. F1000Res 2017;6(598). 10.12688/f1000research.10324.1. PMC5414808 28529719

[30] Nierop PM , Hoppener DJ, Buisman FE, et al. Preoperative systemic chemotherapy alters the histopathological growth patterns of colorectal liver metastases. J Pathol Clin Res 2022;8(1):48-64. 10.1002/cjp2.235. 34480530 PMC8682940

[31] Bilchik AJ , Poston G, Curley SA, et al. Neoadjuvant chemotherapy for metastatic colon cancer: a cautionary note. J Clin Oncol. 2005;23(36):9073-9078. 10.1200/JCO.2005.03.2334. 16361615

[32] Choti MA. Chemotherapy-associated hepatotoxicity: do we need to be concerned?. Ann Surg Oncol. 2009;16(9):2391-2394. 10.1245/s10434-009-0512-7. 19554374

[33] Rubbia-Brandt L , Audard V, Sartoretti P, et al. Severe hepatic sinusoidal obstruction associated with oxaliplatin-based ­chemotherapy in patients with metastatic colorectal cancer. Ann Oncol. 2004;15(3):460-466. 10.1093/annonc/mdh095. 14998849

[34] Vauthey JN , Pawlik TM, Ribero D, et al. Chemotherapy regimen predicts steatohepatitis and an increase in 90-day mortality after surgery for hepatic colorectal metastases. J Clin Oncol. 2006;24(13):2065-2072. 10.1200/JCO.2005.05.3074. 16648507

[35] Pozzo C , Basso M, Cassano A, et al. Neoadjuvant treatment of unresectable liver disease with irinotecan and 5-fluorouracil plus folinic acid in colorectal cancer patients. Ann Oncol. 2004;15(6):933-939. 10.1093/annonc/mdh217. 15151951

[36] Delaunoit T , Alberts SR, Sargent DJ, et al. Chemotherapy permits resection of metastatic colorectal cancer: experience from Intergroup N9741. Ann Oncol. 2005;16(3):425-429. 10.1093/annonc/mdi092. 15677624

[37] Dehal A , Graff-Baker AN, Vuong B, et al. Neoadjuvant Chemotherapy Improves Survival in Patients with Clinical T4b Colon Cancer. J Gastrointest Surg. 2018;22(2):242-249. 10.1007/s11605-017-3566-z. 28933016

